# Residual choledocholithiasis after choledocholithotomy T-tube drainage: what is the best intervention strategy?

**DOI:** 10.1186/s12876-022-02601-6

**Published:** 2022-12-09

**Authors:** Li Zhang, Lu Li, Junfang Yao, Feifei Chu, Yong Zhang, Huili Wu

**Affiliations:** 1 Department of Gastroenterology, Zhengzhou Central Hospital Affiliated to Zhengzhou University, Zhengzhou, 450007 People’s Republic of China; 2 Department of Medical Ultrasonics, Zhengzhou Central Hospital Affiliated to Zhengzhou University, Zhengzhou, 450007 People’s Republic of China

**Keywords:** Residual choledocholithiasis, T-tube sinus tract, Endoscopic retrograde cholangiopancreatography, Choledochoscopy, Clinical effect

## Abstract

**Background:**

The best intervention approach for residual choledocholithiasis after choledocholithotomy T-tube drainage remains controversial, especially during the period of indwelling T tube and the formation of a sinus. The purpose of the study was to estimate the effects of two therapeutic modalities, namely endoscopic retrograde cholangiopancreatography (ERCP) and choledochfiberscope via the T-tube sinus tract (CDS) on residual choledocholithiasis after choledocholithotomy T-tube drainage.

**Methods:**

A total of 112 patients with residual choledocholithiasis after choledochotomy were included in the study, 50 of which underwent ERCP and 62 patients experienced choledochoscopy via the T-tube sinus tract. The primary outcome measures included the success rate of remove biliary stones, T-tube drainage time, and the average length of hospital stay. The secondary objective was to consider incidence of adverse events including cholangitis, bile leakage, T-tube migration, pancreatitis, bleeding and perforation. After hospital discharge, patients were followed up for two years and the recurrence of choledocholithiasis was recorded.

**Results:**

There was no significant difference in the success rate of stone removal between the two groups. Compared to CDS group, T-tube drainage time and the average length of hospital stay was significantly shorter in the ERCP group. The incidence of complications (cholangitis and bile leakage) in the ERCP group was lower than that in the CDS group, but there was no statistically significant difference. When the T-tube sinus tract is not maturation, ERCP was the more appropriate endoscopic intervention to remove residual choledocholithiasis, particularly complicated with cholangitis at this time period.

**Conclusions:**

ERCP is a safe and effective endoscopic intervention to remove residual choledocholithiasis after choledocholithotomy T-tube Drainage without the condition of T-tube sinus tract restriction.

## Background

Cholelithiasis is a common disease throughout the world [1–3]. Cholecystolithiasis combined with choledocholithiasis occurs in 10–15% of patients with cholelithiasis [4–7]. Currently, major treatment options include open cholecystectomy combined with choledocholithotomy, laparoscopy combined with choledochoscope and laparoscopy combined with endoscopic retrograde cholangiopancreatography (ERCP) [8, 9]. The T-tube was routinely placed for at least 7–10 days postoperative [10]. However, T-tube drainage for laparotomy generally takes 6 weeks for sinus tracts maturity [11, 12]. The maturation time of the T-tube sinus tract after laparoscopic surgery was more than 8 weeks [13]. T-tube sinus maturation requires more time in elderly patients, especially complicated with diabetes, low immunity and malnutrition, which may take three months to six months. Kong J et al. recommended that the choledochoscope can be operated through the T-tube sinus after 8 weeks of indwelling the T-tube [14].

Studies have shown that the residual rate of choledocholithiasis on T-tube cholangiography is as high as 20.99–24% at one week after choledochotomy [15]. The residual rate of choledocholithiasis is approximately 6.8%, even if the stones are removed under the visualization of the choledochoscope [16]. The T-tubes were kept in place more 8 weeks after surgery in choledocholithiasis patients with complicated residual stones. Clinical decision still remains controversial in this complex condition, and choledochoscopy via the T-tube sinus tract is currently often used to remove residual stones [17, 18]. However, the operative modality has certain drawbacks [3, 19]. When the T-tube sinus tract is not maturation in the early postoperative period, choledochoscopy via the T-tube sinus tract surgery is incapable to remove residual stones. At this point patients are at risk of shedding and blocking of the T-tube, or iatrogenic cholangitis. In addition, the digestive and gastrointestinal functions of patients are often declined, because a large amount of bile drainage leads to the loss of nutrients [20]. It needed to be emphasized that the patients experienced a high level of psychological stress due to the long-term indwelling of drainage bag [13, 21].

The best treatment strategy for residual stones after choledocholithotomy T-tube drainage needs to be explored. In this study, we retrospectively analyzed the patients who underwent residual choledocholithiasis after choledocholithotomy T-tube Drainage. The clinical efficacy of two endoscopic interventions: Endoscopic retrograde cholangiopancreatography (ERCP) and choledochfiberscope via the T-tube sinus tract were compared. This study provides evidence for the clinical treatment of complicated choledocholithiasis patients.

## Materials and methods

### Research object

**Fig. 1:**
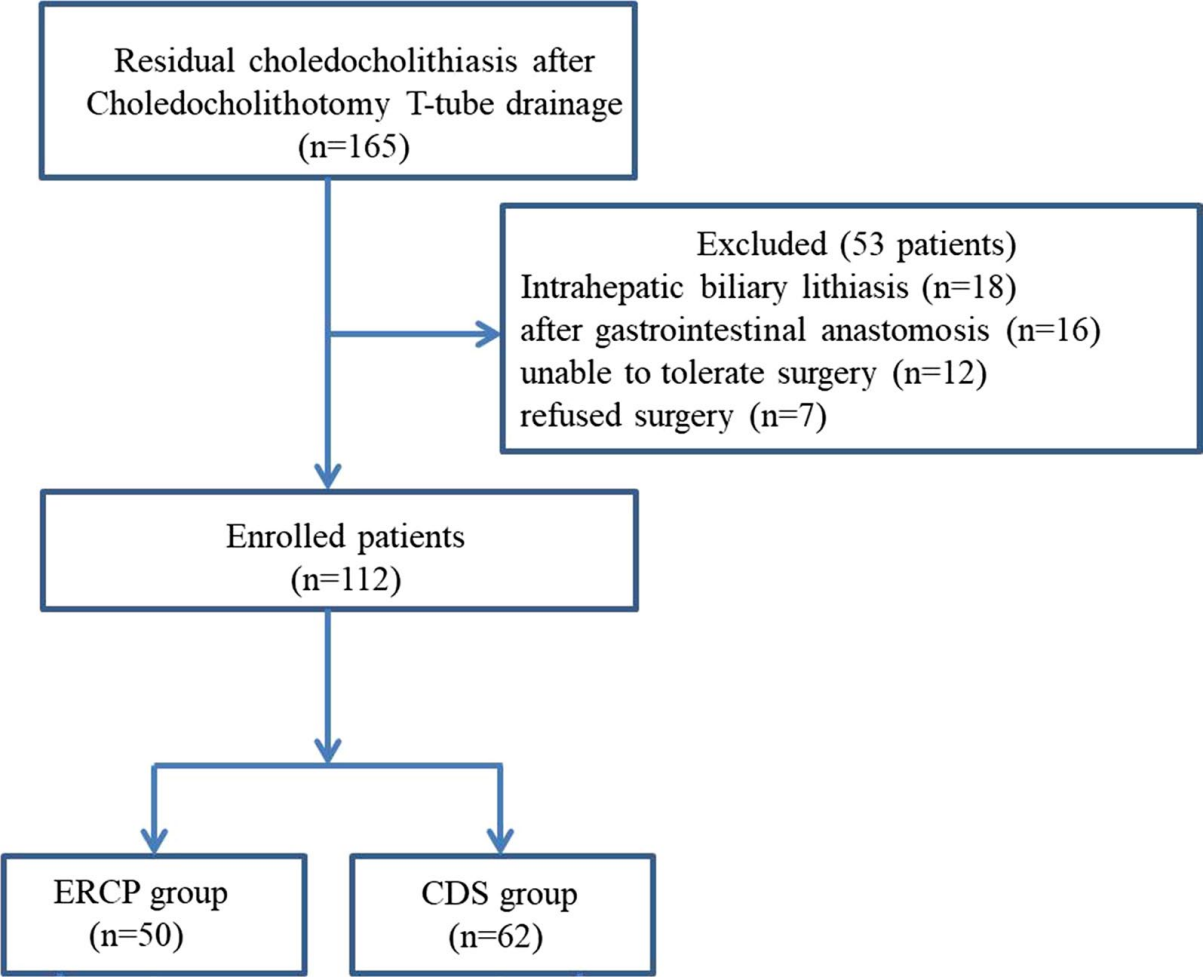
The flow diagram the selection of subjects included in this study. CDS: Choledochfiberscope via the T-tube sinus tract. ERCP: Endoscopic retrograde cholangiopancreatography

This retrospective study was conducted from January 2016 and January 2020 in Zhengzhou Central Hospital Affiliated to Zhengzhou University. After choledochectomy and T-tube drainage, patients diagnosed with residual choledocholithiasis by T-tube cholangiography or magnetic resonance cholangiopancreatography (MRCP) were included in this study. It is worth noting that the enrolled patient's T-tube drainage remains in place at this time. In total, 165 participants were screened out. The exclusion criteria were listed as follows: a) intrahepatic biliary lithiasis, b) after gastrointestinal anastomosis, c) serious cardiac dysfunction and (or) coagulation abnormalities, d) refused surgery. Thus, 112 patients were finally eligible for inclusion in this study. There are 50 patients undergoing endoscopic ERCP lithotripsy treatment. And the 62 patients received choledochoscopy via T-tube sinus. The flowchart of the screening process is shown in Fig. 1. The baseline data of the two groups including surgical modality of choledocholithotomy (laparotomy or laparoscopy), age, gender, size and number of residual stones, combined with underlying diseases are shown in Table 1. This study has been approved by the Medical Ethics Committee of Zhengzhou Central Hospital Affiliated to Zhengzhou University (202202).

**Table 1:** Baseline information of the enrolled patients

	ERCP group	CDS group	*p* value
The median age	65 ± 6.5	68 ± 4.7	0.41
> 60	37	41	
≤ 60	13	21	
Sex(male)	50	62	0.95
male	36	45	
Female	14	17	
Surgical approach	50	62	0.96
Laparotomy	24	30	
Laparoscopic surgery	26	32	
Stone size	50	62	0.88
≥ 1 cm	6	8	
< 1 cm	44	54	
Stone number	50	62	0.83
≥ 2	12	16	
< 2	38	46	
Comorbidities	29	32	
Diabetes	5	7	0.58
Coronary heart disease	8	7	0.46
Chronic obstructive pulmonary disease	4	5	0.99
Hypertension	12	13	0.70

*ERCP* Endoscopic retrograde cholangiopancreatography, *CDS* Choledochfiberscope via the T-tube sinus tract

### Equipment

The equipment used in this study includes fiber cholangioscopes (Olympus CHF-P60), duodenoscope (Olympus TJF260 or JF260V), arch type papillotomy knife (Boston Scientific or Olympus), nasobiliary drainage tube COOK Christmas tree bracket, stone extraction balloon, hydrophilic guidewire, retrieving stone baskets and other related accessories.

### Endoscopic intervention procedures

The ERCP groups were performed as follows. Endoscopic procedures were performed using duodenoscopes. After routine procedures of remove stones, included bile duct cannulation via duodenal papilla, incision of the duodenal papillary muscle, balloon dilation, stone extraction balloon, retrieving stone basket, the nasobiliary drainage tube or plastic stents were kept in place after surgery. After the maturation of the T-tube sinus tract, the T-tube, nasobiliary drainage tube or plastic stents were sequentially extubated.

**Fig. 2:**
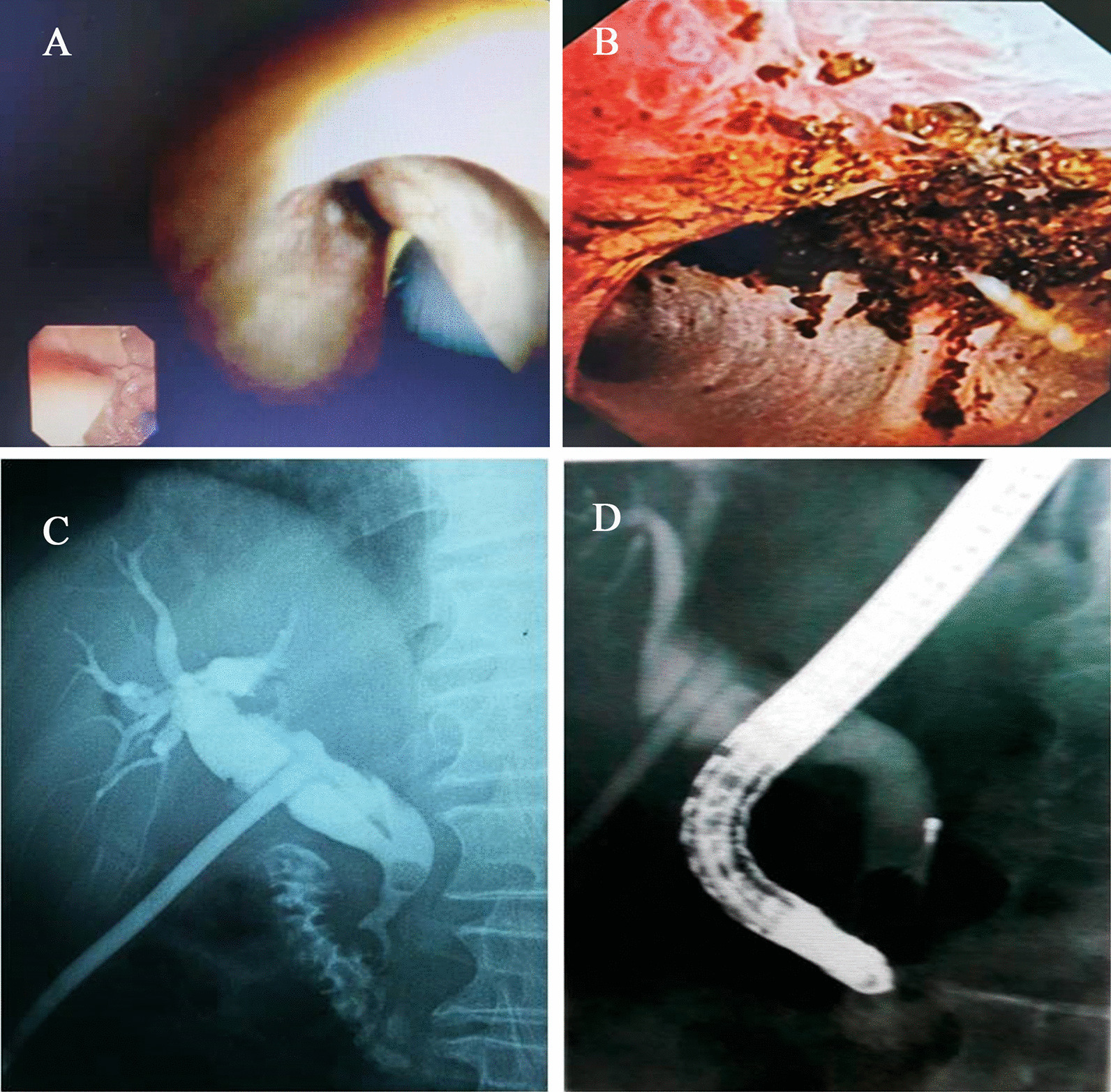
The process of CDS operation and ERCP operation. **A** Choledochoscopy guide wire guide operation. **B** Choledochoscope observation of residual stones in the common bile duct. **C** T tube angiography prompts residual stones in the lower part of the common bile duct. **D** ERCP stone removal basket for stone removal operation. CDS: Choledochfiberscope via the T-tube sinus tract. ERCP: Endoscopic retrograde cholangiopancreatography

The CDS groups were performed as follows. The formation of T-tube sinus tract was evaluated adequately. With the aid of the guide wire, the T tube was removed, and then the straight sheath was inserted. Choledochoscopy via T-tube sinus was performed to remove residual stones. The T tube was pulled out 7–14 days after surgery based on T-tube cholangiogram to show stone-free status (Fig. 2).

### Efficacy evaluation

The primary outcome measures included the success rate of remove biliary stones, T-tube drainage time, and the average length of hospital stay. The secondary objective was to consider incidence of adverse events including cholangitis, bile leakage, T-tube migration, pancreatitis, bleeding and perforation. After discharge, patients were followed up for two years and the recurrence of choledocholithiasis was recorded. The successful of remove stones was defined as complete stone removal confirmed by cholangiography or MRCP over a single surgery session.

### Statistical analysis

Statistical analyses were conducted using the SPSS 20.0 software. The measurement data is expressed as $\overline{x }\pm s$. Student’s t-test was used to compare mean between two groups, and Paired t-test was used to compare data before and after treatment. The counting data were tested by a chi-square test. *P* < 0.05 indicates differences statistically significant.

## Results

### The success rate of stone removal

The success rate of remove residual stones in both groups was 100% after laparotomy. The success rate in ERCP group (96.2%, 25/26) was higher than that of CDS Group after laparoscopic (93.7%, 30/32). However, this observed difference is not to be statistically significant (*P* = 0.68). One patient in ERCP Group had residual stones, and the stones were successfully removed during the second operation. Two patients in the CDS group were converted to ERCP for successful remove stones due to T-tube sinus tract blockage.

### T-tube drainage time and the average length of hospital stay

The time of T-tube drainage in ERCP group were significantly lower in CDS group, whether laparotomy or laparoscopic surgery (Laparotomy, ERCP group *VS* CDS group = 26 ± 5 *VS* 48 ± 6, *P* < 0.01; Laparoscopic, ERCP group *VS* CDS group = 28 ± 6 *VS* 64 ± *5*, *P* < 0.01). These differences were more pronounced after laparoscopy.

The time of the average length of hospital stay in ERCP group were significantly lower in CDS group, whether laparotomy or laparoscopic surgery (Laparotomy, 16 ± 5 *VS* 28 ± 4, *P* < 0.01; Laparoscopic, 13 ± 4 *VS* 26 ± *5*, *P* < 0.01). The results were presented in Table 2.

**Table 2:** Comparison of observation indexes between ERCP group and CDS group

	ERCP group	CDS group	*p* value
*Laparotomy*			
Surgical success rate (%)	100(24/24)	100(30/30)	–
The time of T-tube drainage (days)	26 ± 5	48 ± 6	0.003
Hospital stay (days)	16 ± 5	28 ± 4	0.004
The rate of adverse events (%)	4.2%(1/24)	3.3%(1/30)	0.87
*Laparoscopic*			
Surgical success rate (%)	96.2%(25/26)	93.7(30/32)	0.68
The time of T-tube drainage (days)	28 ± 6	64 ± 5	0.001
Hospital stay (days)	13 ± 4	26 ± 5	0.002
The rate of adverse events (%)	7.7%(2/26)	6.3%(2/32)	0.82

*ERCP* Endoscopic retrograde cholangiopancreatography, *CDS* choledochfiberscope via the T-tube sinus tract

### The occurrence of adverse events

The postoperative complications were not significantly different between the two groups (Laparotomy, ERCP group *VS* CDS group = 4.2% *VS* 3.3%, *P* > 0.05; Laparoscopic, ERCP group *VS* CDS group = 7.7% *VS* 6.3%, *P* > 0.05).

Three patients presented with mild pancreatitis after the ERCP procedure. In terms of complications, there was one case in the laparotomy group and two cases in the laparoscopy group. There were no evident complications of cholangitis and bile leakage from the ERCP procedure.

There were three patients who had cholangitis in CDS group, of which one patient showed with pure cholangitis, one patient presented with obstruction of T tubes sinus tract, and one patient with obstruction of T tubes sinus tract, lacerations in the biliary, bile leakages and slight hemorrhage. There were no fatal cases such as hemorrhage and retroperitoneal perforation in both groups.

### The optimal nodes to remove residual stones

When the sinus is immature, recurrence of residual stones is a formidable problem after choledocholithotomy T-tube drainage. At this point, the application of cholangioscopy is restricted by the temporarily unremovable T tube.

Our results showed that the average length of hospital stay and T tube drainage time for patients with complicated residual stones were shorter in the ERCP group. To remove complex residual stones in the common bile duct, especially, when the patient develops obstructive cholangitis, ERCP can be used for emergency surgery, not limited to T-tube sinus maturity.

### Follow‐up and outcome

Five patients had recurrence during the 24-month follow‐up period, for the record three patients at 18 months and 2 patients at 24 months after surgery. Three patients in the CDS group experienced recurrence of stones. Among these, two patients who underwent laparotomy lithotripsy relapsed at the 18th and 24th months, respectively. One patient who underwent laparoscopic relapsed at 24th months. Fortunately, the recurring sludge was successfully removed by ERCP. During the 18th month follow-up, two patients with recurrence of sludge were recorded in the ERCP group after laparoscopic surgery, and the stones were removed successfully by ERCP again.

## Discussion

ERCP has become a preferred treatment for choledocholithiasis [3, 22]. However, there are controversies about the clinical decision of complex choledocholithiasis, especially the residual stones after choledochectomy and T-tube drainage [23].

Currently, experts generally believe that, after sinus maturity and the removal of T-tube, choledochoscopy possesses multiple advantages, such as convenience, less complications, lower hospitalization cost, accurate imaging diagnosis, and so on [6, 13, 17, 24]. Unfortunately, the removal of residual stones by choledochoscope requires the maturation of the T-tube sinus, which means that the T-tube needs to be indwelled for more than 6–8 weeks. However, the time of T-tube sinus maturity is too long to be tolerated by the patient [10]. In addition, considering the possibility of residual stones during the operation period, the T-tube placed in the abdominal cavity should be thick, short, and straight in order to facilitate the successful removal of the stone in the next operation. However, the process may be considerably more challenging, requiring expert with a large professional experience.

Choledochoscopy via T-tube sinus may produce the following complications: T-tube sinus angular and blockage, drainage tube rupture causes diffuse peritonitis, biliary hemorrhage, biliary penetrance, biliary infections, acute pancreatitis, and so on. In this study, there were three patients with acute cholangitis in CDS group. Among them, two patients who have underwent choledochoscopy were converted to ERCP for successful remove stones due to T-tube sinus tract blockage. In accordance with the findings of Wang et al., T-tube sinus occlusion was successfully restored through X-ray fluoroscopy combined with soft guide wire [25]. However, the manipulation procedure is actually quite difficult. Our study recorded that one patient in CDS- Laparoscopic groups who experienced sinus tear, hemorrhage and biliary peritonitis, was cured after symptomatic, anti-inflammatory, hemostasis and adequate drainage. At a post-operative follow-up 18 months, the patient experienced relapse. Three stones were successfully removed by ERCP.

Several caveats need to be mentioned on choledochoscopy via T-tube sinus. Liu et al. reported that there is a certain success rate and therapeutic effect in the treatment of residual stones in the common bile duct after dilatation of the duodenal papilla via T tube. When the bile duct is significantly dilated, the balloon may push the stone toward the duodenal papilla, causing the stone to escape surgery and fail [26]. According to Zhang et al. the incidence of cholangitis induced by T-tube cholangiography is as high as 8.9% in patients with residual stones after choledochectomy. Acute obstructive suppurative cholangitis caused by obstructed drainage of the T tube or stones located in the proximal bile duct above the T tube will be fatal [27]. In contrast, ERCP attracts people's attention to the treatment of residual stones, because it is not limited to the maturity of the T-tube sinus.

Our retrospective study findings showed that the immediate and long-term complications rates of patients with residual choledocholithiasis treated by conventional ERCP were comparable to those previously reported in conventional cases of ERCP lithotomy. In the present study, two patients presented with bile duct retraction after T-tube drainage. The diameter of the stone is equal or greater than diameter of the lower end of bile duct. For this kind of residual stones, the stones were smoothly removed under the microscope through the balloon dilation of the bile duct opening combined with emergency lithotripsy with a mesh basket.

In addition, one patient with residual stones was completely cleared of choledocholithiasis after two ERCP treatments. During the 18th month follow-up, two patients had recurrence of stones. The recurrence of stones was considered to be related to the angle of the common bile duct, the width of the common bile duct, multiple stones, mechanical lithotripsy, and intestinal fluid reflux [28]. Tsuchiya et al. [29] reported that diagnosis of minute residual stones by micro-bile duct ultrasound and removal by ERCP can reduce the recurrence of common bile duct stones. When combined with difficult cannulation in ERCP, it is worthwhile to try the guide wire through the T tube anteriorly out of the duodenal papilla, and the reverse guide incision for cannulation.

Our team retrospectively analyzed 32 patients with residual choledocholithiasis. The results showed that the ERCP stone removal treatment achieved a perfect success rate and satisfactory safety [30]. In this study, the nasal bile duct was routinely indwelled in patients undergoing ERCP surgery. The supporting effect of the nasobiliary on the common bile duct can reduce the benign stenosis of the common bile duct caused by the removal of the T tube, especially in patients with unobvious common bile duct dilation.

ERCP stone removal treatment requires complicated endoscopic operations. During retrograde imaging, the T-tube drainage should be closed. Due to the indwelling and traction of the T tube, the common bile duct may be distorted [31]. The guide wire may enter the T tube repeatedly when the left and right hepatic ducts are super selected. It requires the surgeon to repeatedly adjust the mirror body to retract the knife in the common bile duct through the guide wire rebound and other operations to repeatedly try super selection [32]. For residual stones in the common bile duct with obvious dilation, the proper application of the stone basket can prevent the stones from escaping. In those patients with indwelling T-tube, the diameter of the lower end of the common bile duct may even be smaller than the diameter of the stone due to the decrease and retraction of the common bile duct pressure. At this time, blindly performing dilation of the duodenal papillary sphincter adds a risk of perforation. It is feasible to perform basket lithotripsy combined with balloon dilatation.

The indwelling nasobiliary drainage should be taken to avoid bending of the bile duct due to T tube traction [33]. The drainage tube is placed in the left intrahepatic bile duct to fully drain and decompress the bile so that the T tube can be removed as soon as possible postoperative. T tube removal time and nasobiliary duct retention time still need to be further clinically studied in such patients after surgery in order to maximize the benefits of patients without complications such as bile leakage.

The T tube drainage time and hospital stay in the ERCP group were significantly shorter than those in the CDS group. The following reasons are considered. T-tube cholangiography was performed at one week after choledocholithotomy, and if there were residual stones in the common bile duct, ERCP surgery was performed immediately. After ERCP treatment, the opening of the duodenal papilla was opened through EST to lead to a smooth flow, and the pressure of the biliary tract was reduced. After ERCP stone removal, complications such as impaired drainage, blockage, and secondary to acute cholangitis do not need to be considered. As bile is drained and excreted normally after ERCP, the patient's physical fitness and appetite improve and accelerate the maturation of the T-tube sinus tract.

## Conclusion

In summary, ERCP is a safe and effective endoscopic intervention to remove residual choledocholithiasis after choledocholithotomy T-tube Drainage without the condition of T-tube sinus tract restriction.

## Data Availability

All data generated during this study are included in this published article.
